# The Week in the Courts

**Published:** 1911-04-22

**Authors:** 


### The Week in the Courts.

### MILK LEGISLATION.

The President of the Local Government Board hopes that it may be possible to introduce the Bill dealing with the supply of milk in the course of the present session.

### BAKING POWDERS AND APPENDICITIS.

Mr. Charles Bathurst asked the President of the Local Government Board whether, in the opinion of the medical advisers of the Board, the prevalence of appendicitis and other intestinal disorders in this country is attributable, either wholly or in part, to the presence of calcium sulphate in baking powders and self-raising flour. Mr. Burns replied that present knowledge did not enable a statement to be made on the subject.

### SLANDER ACTIONS BY MEDICAL PRACTITIONER AND PHARMACIST.

Judgment was given in Dunfermline Sheriff Court in the actions by which Mr. E. Gordon, pharmacist, and Dr. D. F. Sanjana, both of Kelty, sued each other for £500 damages for alleged slander. The Sheriff found that Dr. Sanjana had used words to the effect that Mr. Gordon had fraudulently substituted one medicine for another, and that at a meeting of the local Co-operative Society Dr. Sanjana had used words implying that Mr. Gordon sold adulterated drugs. The Sheriff fixed the damages at £30 and allowed costs on the higher scale. In the action brought by Dr. Sanjana against Mr. Gordon, the Sheriff gave judgment for the defendant with costs.

### CLAIM AGAINST AN "EYE SPECIALIST."

In the Manchester County Court a signalman named Hodson claimed £100 damages from William Mellor, who is not a registered medical practitioner, but describes himself as an "eye specialist." Eight years ago the plaintiff had his left eye cured of a cataract by an operation in the Bolton Infirmary. In 1908 the right eye became affected, but the cataract had not developed to the stage when an operation could be performed. He went to Mellor, who by advertisements claimed "to cure diseases of the eye without resource to the knife." After a lengthy treatment the defendant told Hodson that he would have to go to the surgeon who operated on the other eye. The treatment was by pills and drops, the latter consisting of 98 per cent. of olive oil and 2 per cent. of the extractive matter of pulsatilla. The Judge found for the plaintiff, and awarded £30 damages, with costs on the highest scale.

